# Supplementary material for: Decision-making during obstetric emergencies: A narrative approach
Source: PLoS One. 2022 Jan 26;17(1):e0260277. doi: 10.1371/journal.pone.0260277 (PMC8791468; doi:10.1371/journal.pone.0260277)
Supplement: S1 Appendix — Interview guide used in the rare cases of a stalled conversation. (PDF) [file pone.0260277.s001.pdf]

S1 Appendix. Interview guide. Interview guide used in the rare cases of a stalled conversation.

Beginning - The framework for the conversation and the introductory story is created

1. Can you tell about the case, what you thought and how you felt at the time?
2. Feel free to tell more about…

More exploratory questions

3. Was there anything that surprised you? Was there anything you felt was challenging? Did you feel the need to adapt? Did you have to sacrifice anything during your decisions?
4. … In what way?
5. Feel free to tell more about…

Opportunity for self-reflection concerning the presented case

6. Is there something you wish you had done differently? Why?

Perceptions on respondent's own decision-making process

7. What do you think about your way of making decisions, and how would you describe it? How competent do you perceive yourself to be in decision-making? What does it look like under time pressure?
8. How do you make decisions? Is there any special information you know you are looking for or need (e.g., algorithms, emotions, confirmation from others, thinking out loud, etc.).
