# Supplementary material for: Decision-making during obstetric emergencies: A narrative approach
Source: PLoS One. 2022 Jan 26;17(1):e0260277. doi: 10.1371/journal.pone.0260277 (PMC8791468; doi:10.1371/journal.pone.0260277)
Supplement: S2 Appendix — Intervjuguide, används i de fallen samtalet stannar upp. (DOCX) [file pone.0260277.s002.docx]

**S2 Appendix. Interview guide in Swedish.** Intervjuguide, används i de fallen samtalet stannar upp.

**I början skapas ramen för samtalet och den inledande berättelsen**

1. Kan du återberätta om fallet, hur du tänkte och hur du kände då?
2. Berätta gärna mer om…

**Mera undersökande frågor**

1. Var det något som överraskade dig? Var det något du kände var utmanande? Kände du att du behövde anpassa dig? Fick du offra något vid dina beslut?
2. På vilket sätt?
3. Berätta gärna mer om…

**Möjlighet till självreflektion kring fallet**

1. Är det något som du i efterhand önskar du hade gjort annorlunda? Varför?

**Generell uppfattning av den egna beslutsprocessen**

1. Vad tycker du själv om ditt sätt att fatta beslut, och hur skulle du beskriva det? Hur kompetent uppfattar du dig till att vara i beslutsfattande? Hur ser det ut under tidspress?
2. Hur fattar du beslut? Är det någon särskild information du vet du letar efter eller behöver (exempelvis: algoritmer, känslor, andras bekräftelse, att tänka högt, mm)?
