# Supplementary material for: Decision-making during obstetric emergencies: A narrative approach
Source: PLoS One. 2022 Jan 26;17(1):e0260277. doi: 10.1371/journal.pone.0260277 (PMC8791468; doi:10.1371/journal.pone.0260277)
Supplement: S3 Appendix — List of visual materials chosen by respondents. (DOCX) [file pone.0260277.s003.docx]

**S3 Appendix. List of visual materials.**

List of the works of art in the images chosen by the physicians during the interviews

**Physician 1:**

- Apollo in the forge of Vulcan (*La fragua de Vulcano*), painting by Diego Velázquez, created 1629–1630
- Diana and Actaeon, painting by Titian, created 1556–1559

**Physician 2:**

- The dance (*La danse*), painting by Henri Matisse, created 1909
- The flood (*Le déluge*), painting by Nicolas Poussin, created 1660–1664

**Physician 3:**

- Texan, Portrait of Robert Rauschenberg, artwork by Andy Warhol created 1963
- Portrait of an artist, painting by David Hockney, created 1972
- The dance (*La danse*), painting by Henri Matisse, created 1909

**Physician 4:**

- Highway and byways, painting by Paul Klee, created 1929
- The scream (*Skrik*), painting by Edvard Munch, created 1893

**Physician 5:**

- Grace Coddington wearing ‘The Footer’ mini dress by Mary Quant, fashion photography by David Bailey, created 1967
- The Rondo of the Hours (*L' éternité et la mort*, or *La Ronde des heures*, or *Les Heures*), painting by Xavier Mellery, created 1890

**Physician 6:**

- Les Pieux, painting by Léon Spilliaert, created 1910

**Physician 7:**

- Migrant mother, photography by Dorothea Lange, created 1936
- Duchamp sitting in front of fountain, photography by Julian Wasser, created 1963
- The dance (*La danse*), painting by Henri Matisse, created 1909
- Francis Picabia, photography by Man Ray, created 1922

**Physician 8:**

- Figure on the rocks, painting by Salvador Dali, created 1926

**Physician 9:**

- Judith beheading Holofernes (*Giuditta e Oloferne*), painting by Caravaggio, created 1598-1599 or 1602
- Holy Virgin Mary, painting by Chris Ofili, created 1996
- The water-lily pond (*Le pont japonais sur le bassin aux nymphéas à Giverny*), painting by Claude Monet, created 1899
- Divorce in Moscow, photography by Eve Arnold, created 1966
- The dance (*La danse*), painting by Henri Matisse, created 1909
- The agony in the kitchen, photography by Jessica Todd Harper, created 2012
- The tiger who came to tea, illustration by Judith Kerr from her children’s book with the same name, created 1968

**Physician 10:**

- Dream caused by the flight of a bee around a pomegranate, one second before awakening, painting by Salvador Dali, created 1944
- Venus and Amorini, painting by Salvador Dali, created 1925

**Physician 11:**

- White car crash 19 times, artwork by Andy Warhol, created 1963
- Dali, nude, entranced in the contemplation of five regular bodies metamorphosed in corpuscles, in which suddenly appears Leonardo's "Leda", chromosomatised by the face of Gala, painting by Salvador Dali, created 1954
- Number 5 (red wall), painting by Ad Reinhardt, created 1952
- The lightning field [a photography of a lightning bolt hitting the ground, with a dark sky and seen from a distance], as documented by photographer John Cliett in 1978 and 1979. ‘The lightning field’ is Walter de Maria’s land art work situated in Western New Mexico, created 1977

**Physician 12:**

#### The Blue phantom, painting by Alfred Otto Wolfgang Schulze a.k.a. Wols, created 1951

#### The Cyclops (Le Cyclope), painting by Odilon Redon, created 1914

#### The Doom fulfilled, painting by Edward Burne-Jones, created 1885

- The jungle (*La jungla*), painting by Wilfredo Lam, created 1943

**Physician 13:**

- The iceberg, painting by Frederic Edwin Church, created 1891
- The wounded angel (*Haavoittunut enkeli)*, painting by Hugo Simberg, created 1903

**Physician 14:**

- Galatea of spheres, painting by Salvador Dali, created 1952
- La Mariée mise à nu par ses célibataires, même alias Le Grand Verre, sculpture by Marcel Duchamp, created 1915-1923
- Self-portrait with cigarette (*Selvportrett med sigarett*), painting by Edvard Munch, created 1895
- The persistence of memory, painting by Salvador Dali, created 1931

**Physician 15:**

- Lysistrata Haranguing the Athenian Women, illustration by Aubrey Beardsley, created 1896

**Physician 16:**

- The Apotheosis of Homer, painting by Salvador Dali, created 1944

**Physician 17:**

- Hatstand, table and chair, sculpture set by Allen Jones, created 1969
- The lightning field [a photography of a lightning bolt hitting the ground, with a dark sky and seen from a distance], as documented by photographer John Cliett in 1978 and 1979. ‘The lightning field’ is Walter de Maria’s land art work situated in Western New Mexico, created 1977
- Rán, illustration by Arthur Rackham, created 1911
- The Rhine maidens lament the loss of the Rhinegold, illustration by Arthur Rackham, created 1900
